# Exploring the role of body mass index-adjusted calf circumference within the SARC-CalF screening tool among older patients with cancer

**DOI:** 10.1016/j.jnha.2024.100251

**Published:** 2024-04-26

**Authors:** Maria Karolainy do Nascimento, Jarson Pedro da Costa Pereira, Janaína Oliveira de Araújo, M. Cristina Gonzalez, Ana Paula Trussardi Fayh

**Affiliations:** a Postgraduate Program in Health Sciences, Health Sciences Center, Federal University of Rio Grande do Norte, Natal, RN, Brazil; b Postgraduate Program in Nutrition and Public Health, Department of Nutrition, Federal University of Pernambuco, Recife, PE, Brazil; c Postgraduate Program in Nutrition and Food, Federal University of Pelotas, Pelotas, RS, Brazil; d PesqClin Lab, Onofre Lopes University Hospital, Brazilian Company of Hospital Services (EBSERH), Federal University of Rio Grande do Norte, Natal, RN, Brazil

**Keywords:** Sarcopenia screening, Calf circumference, Body mass index, Cancer, Mortality

## Abstract

**Objectives:**

This study aimed to assess and compare the frequency of positive scores using unadjusted SARC-CalF with the scores derived from SARC-CalF after adjusting calf circumference (CC) for body mass index (BMI). The secondary aim was to assess the prognostic value of SARC-CalF after BMI adjustment, for length of hospital stay (LOS) and mortality.

**Design, Setting, and Participants:**

This secondary analysis of a prospective cohort study, included both outpatients and inpatients of an oncology unit hospital in Brazil.

**Measurements:**

BMI and CC were measured. Patients with excess weight had their CC adjusted for BMI by subtracting 3 cm, 7 cm, and 12 cm from the unadjusted CC values for respective BMI categories. SARC-CalF was used to screen for sarcopenia. Scores ≥11 were indicative of sarcopenia, considering both unadjusted and BMI-adjusted CC values. Clinical outcomes included prolonged LOS and both short- and long-term mortality.

**Results:**

Our study included 206 subjects, with a median age of 69 years, and the majority were males (52.1%). The prevalence of low CC increased from 65% to 84% after BMI adjustment. Positive unadjusted SARC-CalF scores (≥11) were observed in 51% of the population and this prevalence increased to 65% using BMI-adjusted SARC-CalF criteria (≥11). Higher scores on BMI-adjusted SARC-CalF but not unadjusted SARC-CalF were independently associated with prolonged LOS [_adjusted_ HR: 1.26 (1.03–1.53)], and 6-month mortality [_adjusted_ HR: 1.42 (1.07–1.87)]. Both unadjusted and BMI-adjusted SARC-CalF were independently associated with 12-month mortality.

**Conclusion:**

BMI-adjusted SARC-CalF may be a promising strategy to enhance the detection of older patients with cancer and excess weight at risk of sarcopenia, and it may serve a dual role as a prognostic tool, as it was independently associated with prolonged LOS and mortality.

## 1 Introduction

Sarcopenia, marked by the progressive decline in muscle mass, strength, and functionality [1], is a prevalent concern among older adults with cancer. This condition significantly influences treatment effectiveness, impairs quality of life, and adversely affects overall survival rates [[2], [3], [4], [5]]. Assessing sarcopenia within this clinical population is of paramount importance for optimizing their treatment and care [6]. Nevertheless, in clinical practice, the availability or feasibility of utilizing appropriate instruments for assessing muscle mass and strength during treatment can be limited [7,8]. In this context, screening tools for sarcopenia are crucial in early detection and intervention. These tools empower healthcare providers to identify individuals at risk or in the early stages of sarcopenia before a significant functional decline occurs, thus enabling timely intervention and personalized care strategies [9,10].

SARC-F is a simple, validated, and practical tool widely employed in clinical settings to screen for sarcopenia among older individuals [[11], [12], [13]]. Despite being highly recommended, prior studies have indicated its limited efficacy for this purpose, demonstrating a low to moderate sensitivity in sarcopenia diagnosis [[14], [15], [16], [17], [18]]. As a response to this limitation, an improvement to the diagnostic performance of SARC-F was proposed by incorporating calf circumference (CC) as a marker of muscle mass, leading to the development of SARC-CalF [19]. After the inclusion of CC, an increase in the sensitivity was observed compared to relying solely on the SARC-F results [20]. However, an ongoing debate surrounds the impact of body size and adiposity on anthropometric markers such as CC [21].

To mitigate the effects of excess weight on CC, Gonzalez et al. [22] proposed the adjustment of CC according to body mass index (BMI). Nevertheless, the impact and prognostic value of this novel approach, particularly its application to SARC-CalF scores, has not been investigated. Therefore, our study aimed to compare the frequency of positive scores on sarcopenia screening tools using unadjusted SARC-CalF and SARC-CalF after BMI-adjusted CC. Additionally, we aimed to explore the prognostic value of BMI-adjusted SARC-CalF in older patients with cancer.

## 2 Methods

### 2.1 Study design and participants

This is a time-expanded secondary analysis of a prospective cohort [23] involving patients from the Onofre Lopes University Hospital (HUOL) in Rio Grande do Norte, Brazil, conducted between the 2021 and 2023. The study included both genders, and consecutively recruited older adult outpatients and inpatients (≥60 years) from the oncology unit. Participants with incomplete data (height, weight, and calf circumference), had edema/ascites/anasarca or concomitant catabolic diseases (heart failure, acquired immunodeficiency syndrome, inflammatory bowel diseases, chronic kidney disease, non-oncological liver diseases, and tuberculosis) were excluded. All participants provided their written informed consent. The Hospital's Ethics Committee approved this study under the protocol number (CAAE 40045220.7.0000.5292).

### 2.2 Clinical assessment, outcomes and covariates

Sociodemographic and clinical information were obtained from the electronic medical records. Data on age, comorbidities, tumor site and stage, and treatment performed were recorded. Clinical outcomes were assessed based on several parameters. Prolonged length of hospital stay (LOS) was defined as exceeding the median duration of more than 7 days within our study population. Additionally, mortality was evaluated at multiple time points, including in-hospital, 6-month, and 12-month intervals. The Eastern Cooperative Oncology Group Performance Status (ECOG-PS) was employed to assess the functionality of the participants [24].

### 2.3 Anthropometric assessment

Body weight (kg) and height (m) were measured to calculate body mass index (BMI), which was classified under the World Health Organization (WHO) guidelines. Individuals with BMI ≥ 25 kg/m² were classified as excess weight. CC was measured using an inelastic tape (CESCORF®, Brazil) at the greatest prominence of the calf. The leg was positioned at a 90º angle during the assessment. CC was considered low if ≤ 33 cm for women or ≤34 cm for men, as proposed by Barbosa-Silva et al. [25]. For individuals with excess weight, CC was adjusted by subtracting 3 cm, 7 cm, and 12 cm from the unadjusted CC values for respective BMI categories: 25–29.9 kg/m², 30–39.9 kg/m², and ≥40.0 kg/m² [22].

### 2.4 Sarcopenia screening

SARC-CalF contains five domains assessing various activities in addition to CC. Each domain (strength, assistance with walking, rising from a chair, and climbing stairs) is scored from 0 to 2 based on the level of difficulty (0: none, 1: some, 2: a lot or unable to perform). For the falls criterion, 0 indicates no falls, 1 indicates 1–3 falls, and 2 indicates 4 or more falls. [11,19]. The CC item is scored based on the CC cut-off point. If patients exhibit low CC (as previously described), they receive an additional 10 points on the SARC-CalF score. Otherwise, 0 points are assigned. A SARC-CalF score of ≥11 indicates sarcopenia, considering both unadjusted and BMI-adjusted CC.

### 2.5 Statistical analyses

Data were analyzed using the Statistical Package for the Social Sciences (SPSS), version 20.0 (SPSS Inc., Chicago, IL, USA), and MedCalc version 22.0.0.9 software (MedCalc, Mariakerke, Belgium). Normality of the continuous variables was assessed using the Shapiro-Wilk test. Data with normal distribution was described as mean and standard deviation (SD). Non-normal data was described as median and interquartile ranges (IQ). Categorical variables were presented as frequencies (n) and relative percentages (%). Sex- and SARC-CalF-specific comparisons were conducted using the following tests: independent Student “t”, Mann–Whitney U test, Pearson’s χ² or Fisher’s Exact tests, as appropriate. The variance ratio of SARC-CalF scores was assessed using the F-test. Crude and adjusted Cox regression analyses were conducted to assess the association between unadjusted and BMI-adjusted SARC-CalF with outcomes. For statistical adjustment, clinical variables pertaining to cancer prognosis were incorporated based on clinical plausibility. Statistical significance was set at *P* < 0.05 for all analyses.

## 3 Results

**Fig. 1:**
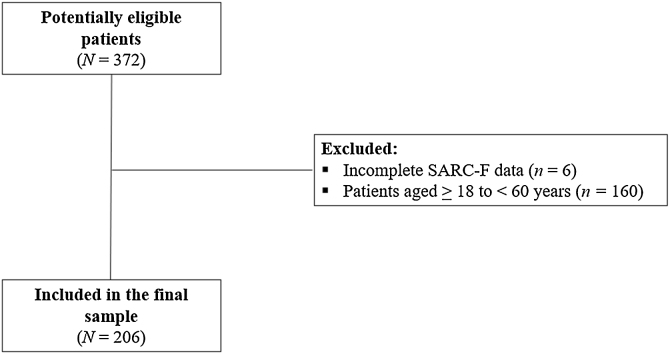
Study flowchart.

Our study initially involved the assessment of 372 patients. However, to strengthen the significance of SARC-CalF, we excluded adults and those with incomplete SARC-F (*n* = 166), resulting in a final sample of 206 older patients, Fig. 1. The median age of the patients was 69 years (IQ: 64.7−75), with a majority being males (52.1%), inpatients (68.4%), and having solid tumors (89.8%). Patients were monitored throughout their hospital stay and up to 12 months post-discharge to evaluate outcomes. The overall mortality incidence during the follow-up period was 41.9% (*n* = 83). Patients’ characteristics are shown in Table 1.

**Table 1:** Characteristics of patients with cancer.

Variables	Total	Males	Females	*P*^a^, ^b^
*N*, %	206	109 (52.1)	98 (47.9)	
Age (years), median and IQ	69 (64.7−75)	69 (64.2−74.7)	70 (64.7−75.2)	0.81
Race/Ethnicity, n (%)				0.39
White	47 (22.8)	23 (21.3)	24 (24.5)	
Black	22 (10.7)	9 (8.3)	13 (13.3)	
Mixed race	137 (66.5)	76 (70.4)	61 (62.2)	
Educational level, n (%)				0.52
Illiterate	34 (17.3)	20 (19.6)	14 (15.1)	
Primary	118 (60.5)	59 (57.8)	59 (63.4)	
Secondary	33 (16.9)	16 (15.7)	17 (18.3)	
Higher	10 (5.1)	7 (6.9)	3 (3.2)	
Setting, n (%)				0.98
Outpatients	65 (31.6)	34 (31.5)	31 (31.6)	
Inpatients	141 (68.4)	74 (68.4)	67 (68.4)	
Reason of hospitalization, n (%)				0.50
Elective	82 (58.2)	45 (60.8)	37 (55.2)	
Non-elective	59 (41.8)	29 (38.2)	30 (44.8)	
Comorbidities, n (%)				
SAH	117 (56.8)	51 (47.2)	66 (67.3)	**0.004**
2DM	56 (27.2)	27 (25.0)	29 (29.6)	0.46
CCI, median and IQ	8 (5−9)	8 (5−9)	7 (5−8)	**0.040**
Type of tumor, n (%)				0.97
Solids	185 (89.8)	97 (89.8)	88 (89.8)	
Hematological	21 (10.2)	11 (10.2)	10 (10.2)	
Tumor site, n (%)				**<0.001**
Hepatobiliary	43 (20.)	18 (16.7)	25 (25.5)	
Colorectal	34 (16.5)	16 (14.8)	18 (18.4)	
Others	32 (15.5)	18 (16.7)	14 (14.3)	
Hematological	20 (9.7)	10 (9.2)	10 (10.2)	
Lung	18 (8.7)	8 (7.4)	10 (10.2)	
Pancreas	17 (8.3)	9 (8.3)	8 (8.2)	
Prostate	16 (7.8)	16 (14.7)	0 (0.0)	
Gastric	14 (6.8)	9 (8.3)	5 (5.1)	
Breast	8 (3.9)	0 (0.0)	8 (8.2)	
Kidney	4 (1.9)	4 (3.7)	0 (0.0)	
TNM stage, n (%)				0.09
I	6 (2.9)	4 (3.7)	2 (2.1)	
II	26 (12.7)	11 (10.2)	15 (15.5)	
III	45 (22.0)	17 (15.7)	28 (28.9)	
IV	86 (42.0)	50 (46.3)	36 (37.1)	
No information	42 (20.5)	26 (24.1)	16 (16.5)	
Treatment modalities, n (%)				
Chemotherapy	80 (39.0)	47 (58.8)	33 (33.7)	0.13
Radiotherapy	2 (1.0)	2 (1.9)	0 (0.0)	0.18
Chemotherapy + radiotherapy	18 (8.7)	9 (8.3)	9 (9.2)	0.83
ECOG-PS scale, n (%)				0.11
0−1	94 (45.6)	55 (50.9)	39 (39.8)	
≥2	112 (54.4)	53 (49.1)	59 (60.2)	
Outcomes, n (%)				
Prolonged LOS	70 (49.6)	37 (50.0)	33 (49.3)	0.93
In-hospital mortality	32 (22.7)	17 (23.0)	15 (22.4)	0.93
6-month mortality	71 (36.0)	45 (43.3)	26 (28.0)	**0.025**
12-month mortality	83 (41.9)	49 (47.1)	34 (36.2)	0.12

Abbreviations: 2DM: type 2 diabetes mellitus; CCI: charlson comorbidity index; ECOG-PS: eastern oncology group performance status; IQ: interquartile range; LOS: length of stay; SAH: systemic arterial hypertension; Sample size: (*n* = 205) for variables chemotherapy, and TNM stage; educational level (*n* = 195); 6-month mortality (*n* = 197); LOS, and in-hospital mortality (*n* = 141), 12-month mortality (*n* = 198).

Bold is used to emphasize significant *P* values.

a Pearson’s χ² or Fisher’s exact test.

b Mann–Whitney U test.

**Fig. 2:**
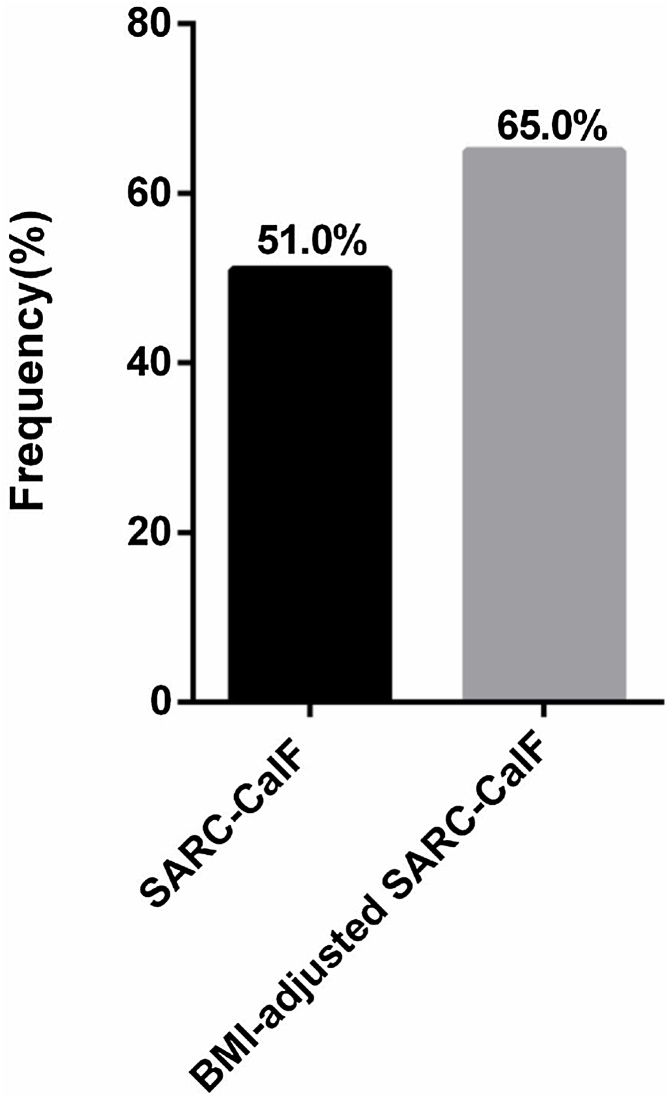
Frequency of positive SARC-CalF with CC adjusted or not by BMI. CC: calf circumference; BMI: body mass index.

Table 2 shows comparisons between anthropometric and screening of sarcopenia between genders. Excess weight was presented in 36.4% (*n* = 75) of the sample and had their CC BMI-adjusted. BMI ≥ 25 was significantly higher in females compared to males (*P* = 0.007). In contrast, the frequency of low unadjusted CC was significantly higher in males compared to females (*P* = 0.010). After adjustment for BMI, the frequency of low CC increased from 65% (*n* = 134) to 84% (*n* = 173). Positive unadjusted SARC-CalF scores (≥11) were found in 51.0% (*n* = 105). After BMI-adjusted CC, the frequency of positive SARC-CalF (≥11) increased to 65.0% (*n* = 134) within our population. Fig. 2 visually depicts the frequency of positive SARC CalF before and after BMI adjustment to CC. Significant variances in scores from unadjusted SARC-CalF compared to BMI-adjusted SARC-CalF were identified (variance ratio: 1.40, *P* = 0.016).

**Table 2:** Morphological characteristics of patients with cancer (*N* = 206).

Variables	Total	Males	Females	*P*^a^, ^b^
BMI (kg/m^2^)				**0.007**^a^
Overweight (≥25), n (%)	54 (26.1)	21 (19.3)	33 (33.7)	
Obesity (≥30), n (%)	19 (9.2)	5 (4.6)	14 (14.3)	
Obesity (≥40), n (%)	2 (1.0)	1 (0.9)	1 (1.0)	
CC (cm)	31.5 (29.2−34.9)	31 (29−35)	32 (30−35)	0.17 b
Low, n (%)	134 (65.0)	79 (73.1)	55 (56.1)	**0.010**^a^
BMI-adjusted CC (cm)	30.6 (28−32)	31 (29−32.3)	30.5 (28−32)	0.13 b
Low, n (%)	173 (84.0)	94 (86.1)	80 (81.6)	0.38 b
SARC-CalF	11 (4.8−14.0)	11 (7−13)	11 (4−14)	
Positive (≥11), n (%)	105 (51.0)	55 (50.9)	50 (51.0)	0.99 b
BMI-adjusted SARC-CalF	11 (10−15)	11 (10−14)	13 (10−15)	
Positive (≥11), n (%)	134 (65.0)	61 (58.5)	73 (74.5)	**0.007**^a^

Abbreviations: BMI: body mass index (kg/m^2^); CC: calf circumference (cm).

Bold is used to emphasize significant *P* values.

a Pearson’s χ² or Fisher’s exact test (absolute frequency and relative %).

b Mann–Whitney U test (median and interquartile range).

Table 3 provides a comparative analysis between patients with and without risk scores by SARC-CalF, employing both unadjusted and BMI-adjusted forms. Patients scoring ≥ 11 exhibited tendencies towards advanced age, significantly higher comorbidity burden (CCI), poorer functional status (ECOG-PS), as well as notably higher 6- and 12-month mortality rates. These observations were consistent across both approaches of SARC-CalF. Among patients with excess weight, Cox regression analyses demonstrated that, except 12-month mortality, unadjusted SARC-CalF wasn’t associated with any clinical outcomes – neither in crude or adjusted models. Unadjusted SARC-CalF was independently associated with 12-month mortality (_adj_ HR: 2.26 [95% CI 1.38–3.71]). After adjusting CC for BMI, SARC-CalF emerged as an independent predictor of prolonged LOS and both 6-month and 12-month mortality (Table 4).

**Table 3:** Comparison of patients with cancer (*N* = 206) with and without risk scores by SARC-CalF, utilizing both unadjusted and BMI-adjusted forms.

	Unadjusted SARC-CalF			BMI-adjusted SARC-CalF		
Variables	Score <11	Score ≥ 11	*P*^a^, ^b^	Score <11	Score ≥ 11	*P*^a^, ^b^
*N*, %	101 (49.0)	105 (51.0)		72 (35.0)	134 65.0)	
Age (years), median and IQ	68 (64−73)	70 (70−76)	0.05	68 (64−73)	70 (65−76)	0.05
Setting, n (%)			**<0.001**			**<0.001**
Outpatients	45 (44.6)	20 (19.0)		34 (47.2)	31(23.1)	
Inpatients	56 (55.4)	85 (81.0)		38 (52.8)	103 (76.9)	
CCI, median and IQ	6 (4−9)	8 (6−9)	**0.003**	6 (4−9)	8 (5−9)	**0.023**
Type of tumor, n (%)			0.29			0.05
Solids	93 (92.1)	92 (87.6)		69 (95.8)	116 (86.6)	
Hematological	8 (7.9)	13 (12.4)		3 (4.2)	18 (13.4)	
TNM staging, n (%)			0.18			0.09
I	5 (5.0)	1 (1.0)		4 (5.6)	2 (1.5)	
II	16 (15.8)	10 (9.6)		11 (15.3)	15 (11.3)	
III	24 (23.8)	21 (20.2)		20 (27.8)	25 (18.8)	
IV	38 (37.6)	48 (46.2)		27 (37.5)	59 (44.4)	
No information	18 (17.8)	24 (23.1)		10 (13.9)	32 (24.1)	
Treatment modalities, n (%)						
Chemotherapy	41 (40.6)	39 (37.5)	0.65	32 (44.4)	48 (36.1)	0.24
Radiotherapy	1 (1.0)	1 (1.0)	0.74	1 (1.4)	1(0.7)	0.57
Chemotherapy + radiotherapy	14 (13.9)	4 (3.8)	**0.013**	11 (15.3)	7 (5.2)	**0.015**
ECOG-PS scale, n (%)			**<0.001**			**<0.001**
0−1	59 (58.4)	35 (33.3)		47 (65.3)	49 (35.1)	
≥2	42 (58.4)	70 (66.7)		25 (34.7)	87 (64.9)	
Outcomes, n (%)						
Prolonged LOS	29 (51.8)	41 (48.2)	0.68	20 (52.6)	50 (48.5)	0.66
In-hospital mortality	11 (19.6)	21 (24.7)	0.48	6 (15.8)	26 (25.2)	0.23
6-month mortality	23 (23.0)	48 (49.5)	**<0.001**	15 (21.1)	56 (44.4)	**0.001**
12-month mortality	27 (27.3)	56 (56.6)	**<0.005**	17 (23.2)	66 (52.0)	**<0.005**

Abbreviations: 2DM: type 2 diabetes mellitus; BMI: body mass index; CCI: charlson comorbidity index; ECOG-PS: eastern oncology group performance status; IQ: interquartile range; LOS: length of stay; SAH: systemic arterial hypertension; Sample size: (*n* = 205) for variables chemotherapy, and TNM stage; educational level (*n* = 195); 6-month mortality (*n* = 197); LOS, and in-hospital mortality (*n* = 141), 12-month mortality (*n* = 198).

Bold is used to emphasize significant *P* values.

a Pearson’s χ² or Fisher’s exact test.

b Mann–Whitney U test.

**Table 4:** Cox regression analysis: Associations between unadjusted and BMI-adjusted SARC-CalF and clinical outcomes among patients with cancer and excess weight.

Variables	HR (95% CI)	*P*
	**Prolonged LOS**	
SARC-CalF	1.02 (0.95–1.10)	0.51
Adjusted model^a^	0.64 (0.35–1.19)	0.16
BMI-adjusted SARC-CalF	1.05 (0.97–1.14)	0.21
Adjusted model^a^	1.26 (1.03–1.53)	**0.025**
	**In-hospital mortality**	
SARC-CalF	1.00 (0.87–1.14)	0.96
Adjusted model^a^	0.91 (0.38–2.19)	0.84
BMI-adjusted SARC-CalF	1.02 (0.89–1.17)	0.79
Adjusted model^a^	1.88 (0.83–4.26)	0.13
	**6-month mortality**	
SARC-CalF	1.04 (0.97–1.11)	0.24
Adjusted model^b^	1.80 (0.94–1.03)	0.08
BMI-adjusted SARC-CalF	1.04 (0.97–1.12)	0.23
Adjusted model^b^	1.42 (1.07–1.87)	**0.014**
	**12-month mortality**	
SARC-CalF	2.64 (1.67–4.18)	**<0.001**
Adjusted model^b^	2.26 (1.38–3.71)	**0.001**
BMI-adjusted SARC-CalF	2.64 (1.55–4.50)	**<0.001**
Adjusted model^b^	1.99 (1.13–3.52)	**0.018**

Abbreviations: BMI: body mass index; LOS: length of hospital stay.

Bold is used to emphasize significant *P* values.

a Models were adjusted for age, gender, reason of hospitalization (elective vs. non-elective), tumor site, TNM staging, functional performance (The Eastern Cooperative Oncology Group Performance Status (ECOG-PS) and Charlson comorbidity index.

b Models were adjusted for age, gender, tumor site, TNM staging, functional performance (The Eastern Cooperative Oncology Group Performance Status (ECOG-PS) and Charlson comorbidity index.

## 4 Discussion

To the best of our knowledge, this is the first study to address the BMI-adjusted CC in SARC-CalF scores, not only in patients with cancer but also in any other clinical condition. Our main findings showed that adjusting CC for BMI significantly increased the frequency of positive scores in SARC-CalF. Moreover, notable variations in scores were observed between unadjusted SARC-CalF when compared to what we conveniently term "BMI-adjusted SARC-CalF”. Lastly, BMI-adjusted SARC-CalF was an independent predictor of prolonged LOS and long-term mortality (6- and 12-months).

Our findings indicate that BMI-adjusted SARC-CalF may be a particularly strong or more sensitive predictor of shorter-term adverse outcomes, as it was the only variable associated with LOS and 6-month mortality. However, when assessing 12-month mortality, both unadjusted and BMI-adjusted SARC-CalF were independently linked to this outcome. We hypothesize that higher scores in SARC-CalF may effectively predict adverse events for long-term outcomes, irrespective of BMI adjustments. This could be because sarcopenia risk, as assessed by SARC-CalF, reflects not just muscle mass (marked by CC) but also muscle function and functional capacity, which are important factors in long-term health outcomes and mortality risk. Therefore, whether adjusted for BMI or not, SARC-CalF remains a valuable tool in predicting adverse events over the longer term. Ururi-Cupi et al. [26] conducted a comprehensive two-year follow-up analysis on the mortality risk among 922 older men with cancer in Peru. Their results unveiled a sarcopenia risk of 45.7% using SARC-CalF criteria. The study demonstrated a noteworthy association between sarcopenia risk, using unadjusted SARC-CalF, and an increased probability of mortality [26].

The results of our study raise critical insights, underscoring the significance of adjusting CC for BMI in patients with excess weight as a critical step to screen for sarcopenia using SARC-CalF. It is well established that excess body fat may obscure anthropometric markers of muscle mass, including CC [27]. For example, when SARC-CalF was initially proposed, the authors emphasized caution when applying it to individuals with excess weight, as they are unlikely to have CC values below the specified cutoffs [19]. In this context, our results indicate that BMI-adjusted SARC-CalF could serve as a practical alternative to mitigate biases related to body weight.

Our group recently demonstrated the prognostic significance of BMI-adjusted CC in a hospitalized population. Individuals with low BMI-adjusted CC exhibited significantly longer LOS [28]. This finding is further emphasized, demonstrating the prognostic significance of BMI-adjusted CC in tools like SARC-CalF, capable of predicting both LOS and mortality. Given the prognostic significance associated with this tool, our results underscore that BMI-adjusted SARC-CalF stands out as a valuable enhancer in prognostic assessments for clinical individuals carrying excess weight. This is particularly relevant, especially considering that unadjusted SARC-CalF did not show any association with clinical outcomes up to 6 months within our cohort.

Despite the novel findings on BMI-adjusted SARC-CalF, previous studies have demonstrated the predictive value of SARC-CalF in populations with cancer [26,29]. Lu et al. [29] highlighted that SARC-CalF exhibited the most robust predictive value among individuals with gastric cancer when compared to other sarcopenia screening tools. Furthermore, Nanri et al.'s study [30] revealed an association between SARC-CalF and a poor functional prognosis in older patients with orthopedic problems. In contrast, Rodrigues et al. [31] found no predictive value for SARC-CalF in a hospitalized population. Nevertheless, we acknowledge the disparities in the studies. The study by Rodrigues et al. [31] encompassed individuals over 18 years, constituting more than half of the sample, potentially obscuring the prognostic significance of SARC-CalF. It is plausible that the SARC questionnaire may be more suitable for older individuals [[1], [2], [3]].

While a global consensus on the definition of sarcopenia, particularly in clinical populations with cancer, remains elusive, the Revised European Working Group on Sarcopenia [1] emphasizes the importance of incorporating muscle mass in sarcopenia diagnosis. In alignment with this, the SARC-CalF, as proposed by Barbosa-Silva [19], introduced CC to the SARC-F as an anthropometric marker of muscle mass. Although SARC-CalF has shown to double the sensitivity in sarcopenia screening [[17], [18], [19]], we posit that a BMI-adjusted SARC-CalF could further enhance sensitivity, particularly among patients with excess weight, thus reducing the likelihood of masking conditions like sarcopenic obesity. Nonetheless, we acknowledge the need for additional studies to investigate and validate our hypothesis thoroughly.

This study has limitations to be acknowledged. The relatively small sample size, particularly regarding patients with excess weight by BMI, may constrain the generalizability of our findings. The broad distribution of cancer diagnoses may not adequately represent the entire spectrum, posing an additional limitation. Future studies should involve larger samples of individuals with excess weight to validate the diagnostic and prognostic significance of BMI-adjusted SARC-CalF in both clinical and healthy populations. On a positive note, our novel study opens avenues for future research and clinical application, showcasing BMI-adjusted SARC-CalF as a potentially enhanced tool for screening sarcopenia among populations with excess weight.

## 5 Conclusion

Our study suggests that utilizing BMI-adjusted SARC-CalF can be a practical approach to better identify older patients with cancer and excess weight at risk of sarcopenia. Furthermore, this approach may serve as a prognostic tool, demonstrating associations with prolonged LOS and mortality, surpassing the short-term predictive power of the unadjusted SARC-CalF. Our novel contribution represents an initial step in exploring the diagnostic and prognostic potential of BMI-adjusted SARC-CalF.

## Author contribution

MKN, JPCP, and APTF contributed to the conception and design of the research; MKN and JOA acquired the data; JPCP, APTF, and MCG contributed to the data analysis. MKN, JPCP, JOA, and APTF wrote the manuscript. APTF and MCG critically revised the manuscript. All the authors critically reviewed, interpreted and approved the final version of the manuscript.

## Funding statement

This study was partially funded by the Coordenação de Aperfeiçoamento de Pessoal de Nível Superior (CAPES), Brazil (Finance Code 001) and the Brazilian National Council for Scientific and Technological Development (CNPq). APTF and MCG received a productivity scholarship from CNPq. The supporting sources have no involvement or restrictions on this publication.

## Ethics declaration

The study was conducted according to the guidelines of the Declaration of Helsinki and its later amendments. All participants provided their written informed consent. The Hospital's Ethics Committee approved this study under the protocol number (CAAE 40045220.7.0000.5292).

## Conflict of interest

APTF reports receiving grant for research from Prodiet Medical Nutrition. MCG has received honoraria and/or paid consultancy from Abbott Nutrition, Nutricia, and Nestlé Health Science Brazil.

## Availability of data and materials

Upon reasonable request.
